# Supplementary material for: A comprehensive temporal patterning gene network in Drosophila medulla neuroblasts revealed by single-cell RNA sequencing
Source: Nat Commun. 2022 Mar 10;13:1247. doi: 10.1038/s41467-022-28915-3 (PMC8913700; doi:10.1038/s41467-022-28915-3)

Supplementary Figure 1

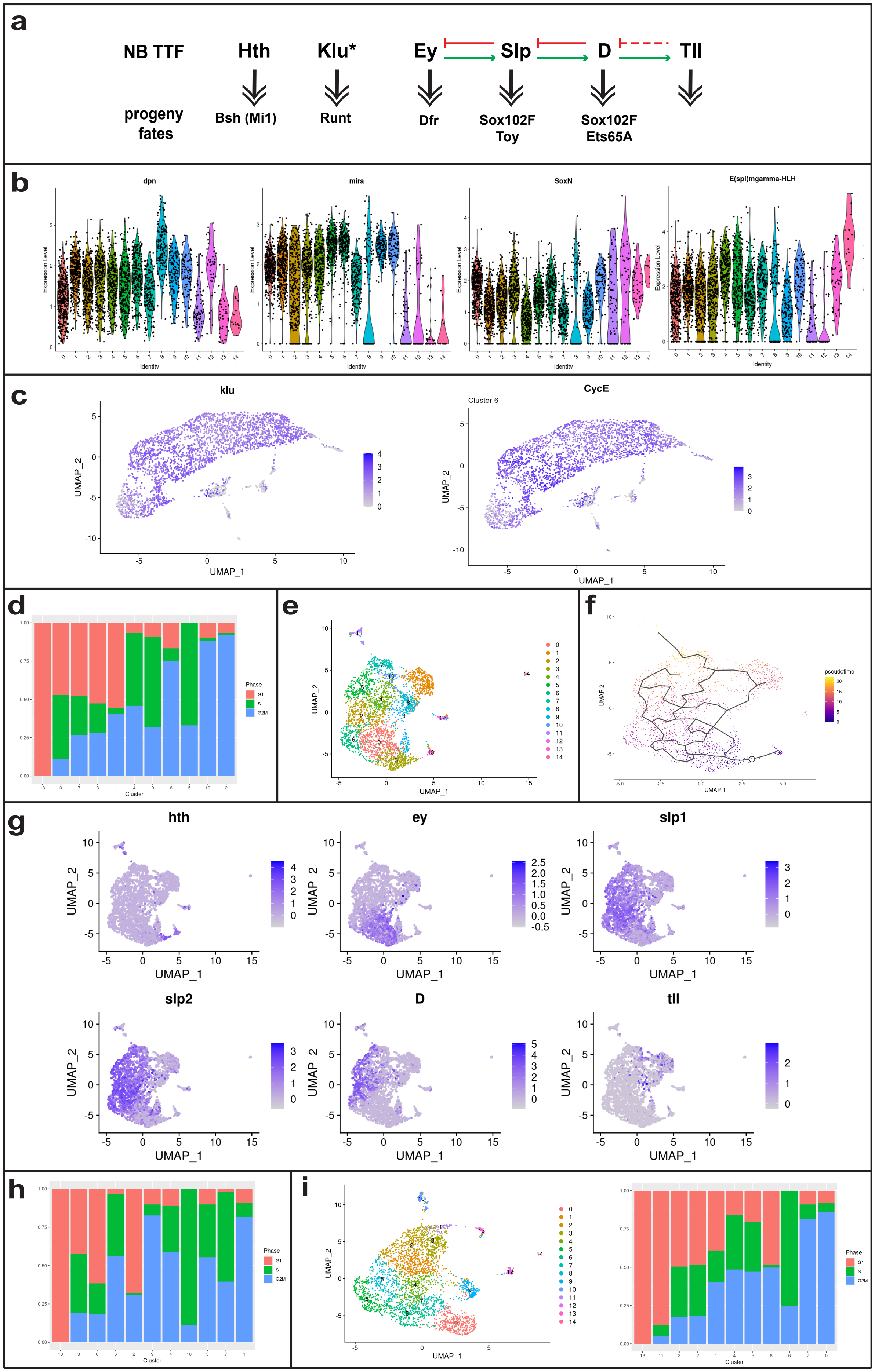

Supplementary Figure 1. Preliminary analysis of scRNA-seq data. (a) A model showing what was known about the medulla TTF cascade before this work. (b) Violin plots showing the expression of marker genes in cells of each cluster. Most cells in clusters 8, 11,12 and 14 have very low level of Mira expression, and are regarded as outlier clusters. Cluster 13 cells are transitioning from NE to NB, and also do not express Mira. (c) The expression patterns of Klu and CycE visualized on UMAP plots. (d) The fraction of cells, within each cluster found in our original analysis without regressing out cell cycle effects, in different phases of the cell cycle. The clusters have been ordered according to the median pseudotime of the cells in each cluster, from smallest to largest. (e-h) Re-analysis of the data with the S phase and G2M phase scores calculated by Seurat (S.Score and G2M.Score) regressed out. (e) Clusters resulted from the re-analysis. (f) The estimated pseudotime trajectories after removing outlier clusters 8, 11, 12, and 14 . (g) Expression patterns of known TTFs visualized on UMAP plots. (h) Fraction of cells within each cluster in different phases of the cell cycle. Cluster ordering was manually modified according to the temporal ordering of the known TTFs. (i) Clusters resulted from re-analysis with regressing out cell cycle genes using the alternate workflow developed by Satija Lab, and the fraction of cells within each cluster in different phases of the cell cycle.

Supplementary Figure 2

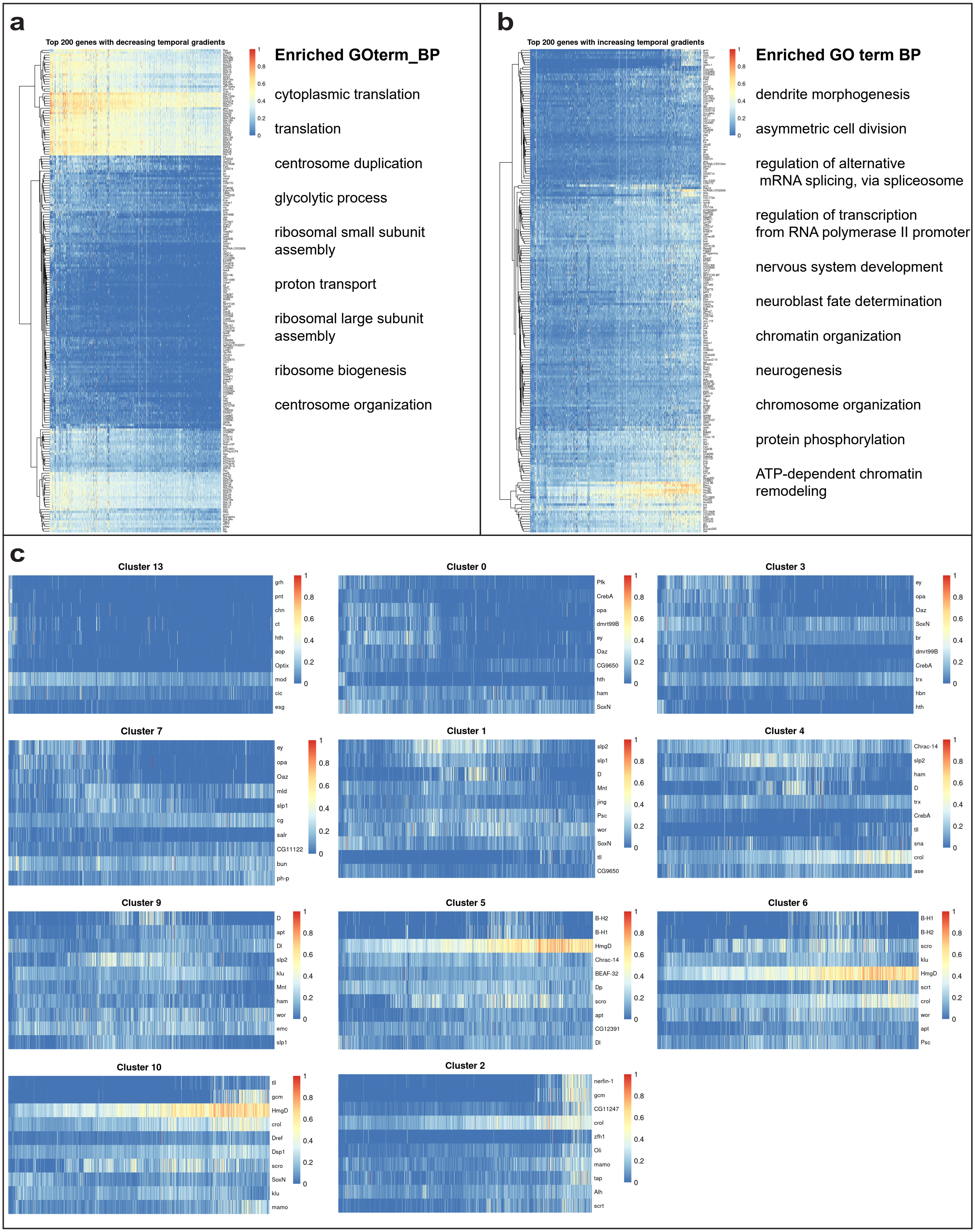

Supplementary Figure 2. Analysis of genes that show a temporal gradient of expression and TFs differentially expressed in each cluster. In all heatmaps, the expression levels are visualized as a percentage of the maximum observed expression across all cells. (a) Heatmaps showing the top 200 genes with decreasing temporal gradients across the pseudotime. Using the Functional Annotation Tool from the DAVID Bioinformatics Resources 6.8, these genes are enriched in the GO terms related to translation and metabolism. For details refer to Supplementary Table 2. (b) Heatmaps showing the top 200 genes with increasing temporal gradients across the pseudotime. These genes are enriched in the GO terms related to the regulation of gene expression, neural development, and signal transduction among others. For details refer to Supplementary Table 3. (c) Heatmaps showing the top 10 differentially expressed TFs for each NB cluster. The clusters are ordered according to their positions along the pseudotime.

Supplementary Figure 3

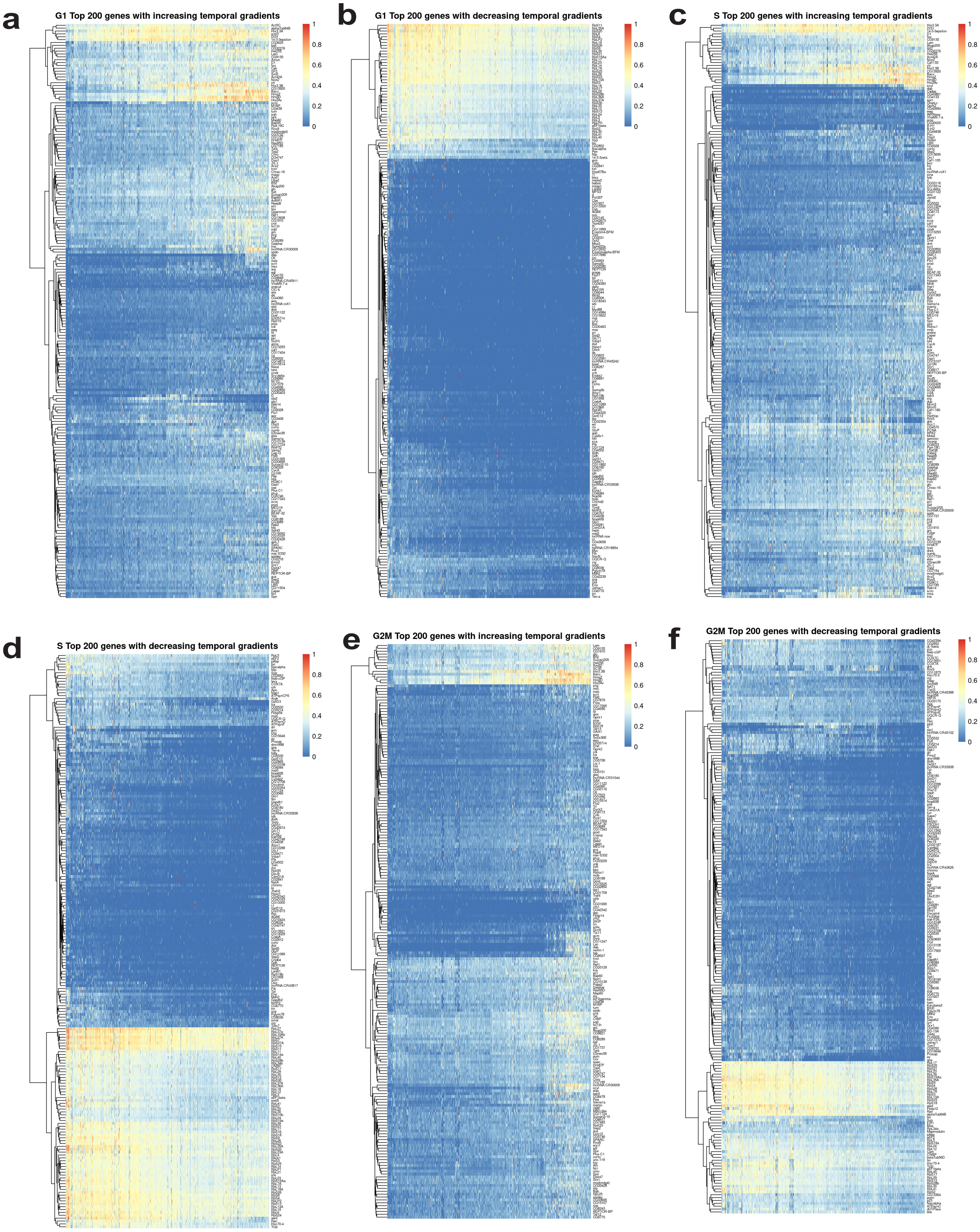

g

	Overlap in top 200 genes positively correlated with pseudotime	Overlap in top 200 genes negatively correlated with pseudotime
G1 phase	129	102
S phase	135	164
G2M phase	158	133

Supplementary Figure 3. Analysis of genes that show a temporal gradient of expression in different phases of the cell cycle. (a,b) In G1 phase cells, heat map showing top 200 genes with increasing (a) or decreasing (b) temporal gradients. (c,d) In S phase cells, heat map showing top 200 genes with increasing (c) or decreasing (d) temporal gradients. (e,f) In G2/M phase cells, heat map showing top 200 genes with increasing (e) or decreasing (f) temporal gradients. (g) A table showing the number of genes that are common between the top 200 genes that have increasing or decreasing gradients within each phase and the top 200 genes from our original gradient analysis with all cells combined.

Supplementary Figure 4

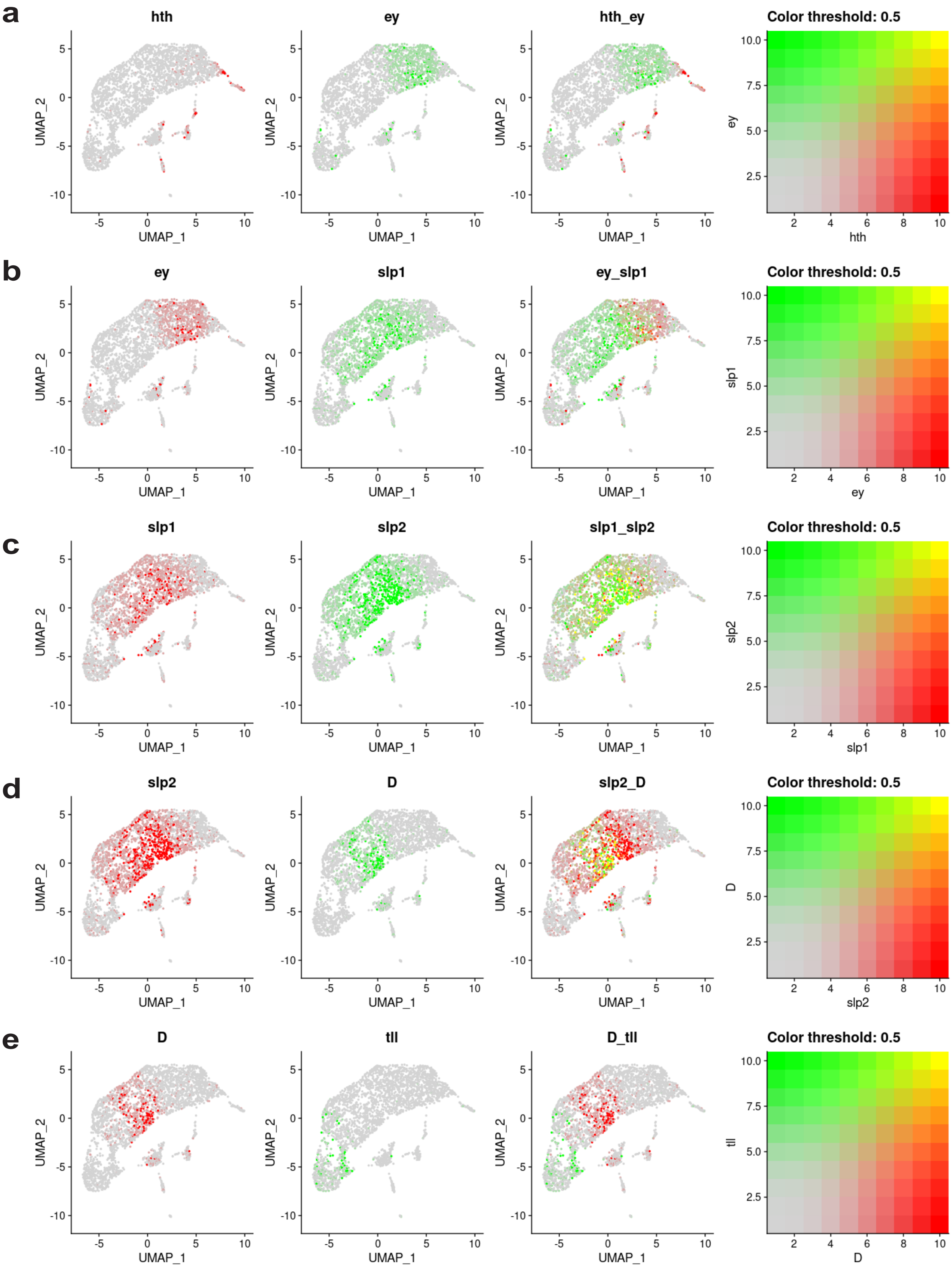

Supplementary Figure 4. UMAP plots of pairs of known TTFs. (a) UMAP plots of hth (red) and ey (green) revealed a gap between hth and ey. (b) UMAP plots of ey (red) and slp1 (green) showed significant partial overlap between the two neighboring TTFs. (c) UMAP plots of slp1(red) and slp2 (green) showed almost complete overlap between the two homologous genes. (d) UMAP plots of slp2 (red) and D (green) showed significant partial overlap between the two neighboring TTFs. (e) UMAP plots of D (red) and Tll (green) revealed a gap between these two stages.

Supplementary Figure 5

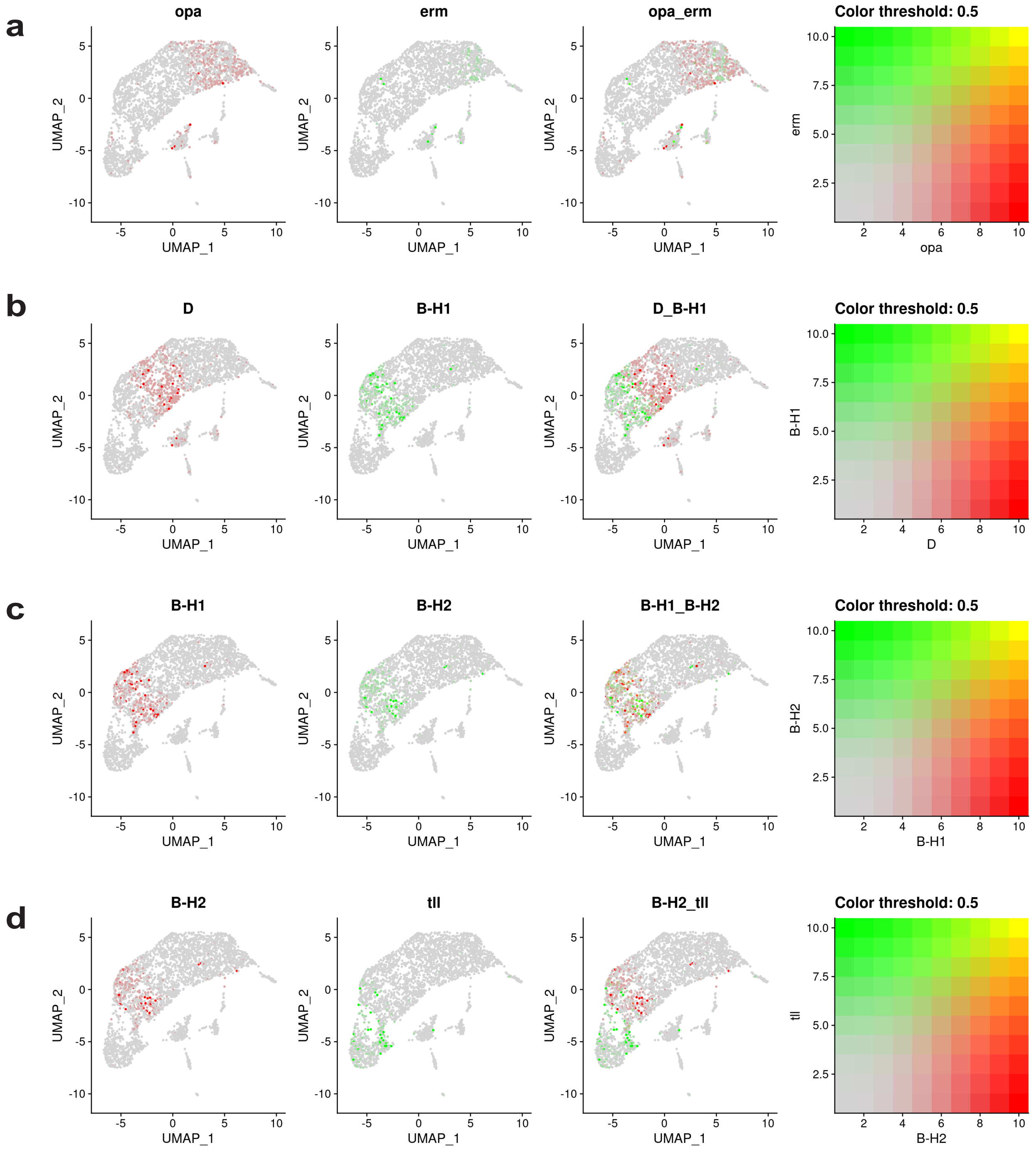

Supplementary Figure 5. UMAP plots of pairs of previously unknown and known TTFs. (a) UMAP plots of opa (red) and erm (green) showed that erm transcripts are present in the gap between the two groups of Opa expressing neuroblasts. (b-d) UMAP plots of D (red) and B-H1 (green) (b), B-H1(red) and B-H2 (green) (c), B-H2 (red) and Tll (green) (d), showed that B-H1 and B-H2 fill the gap between D and Tll stages.

Supplementary Figure 6

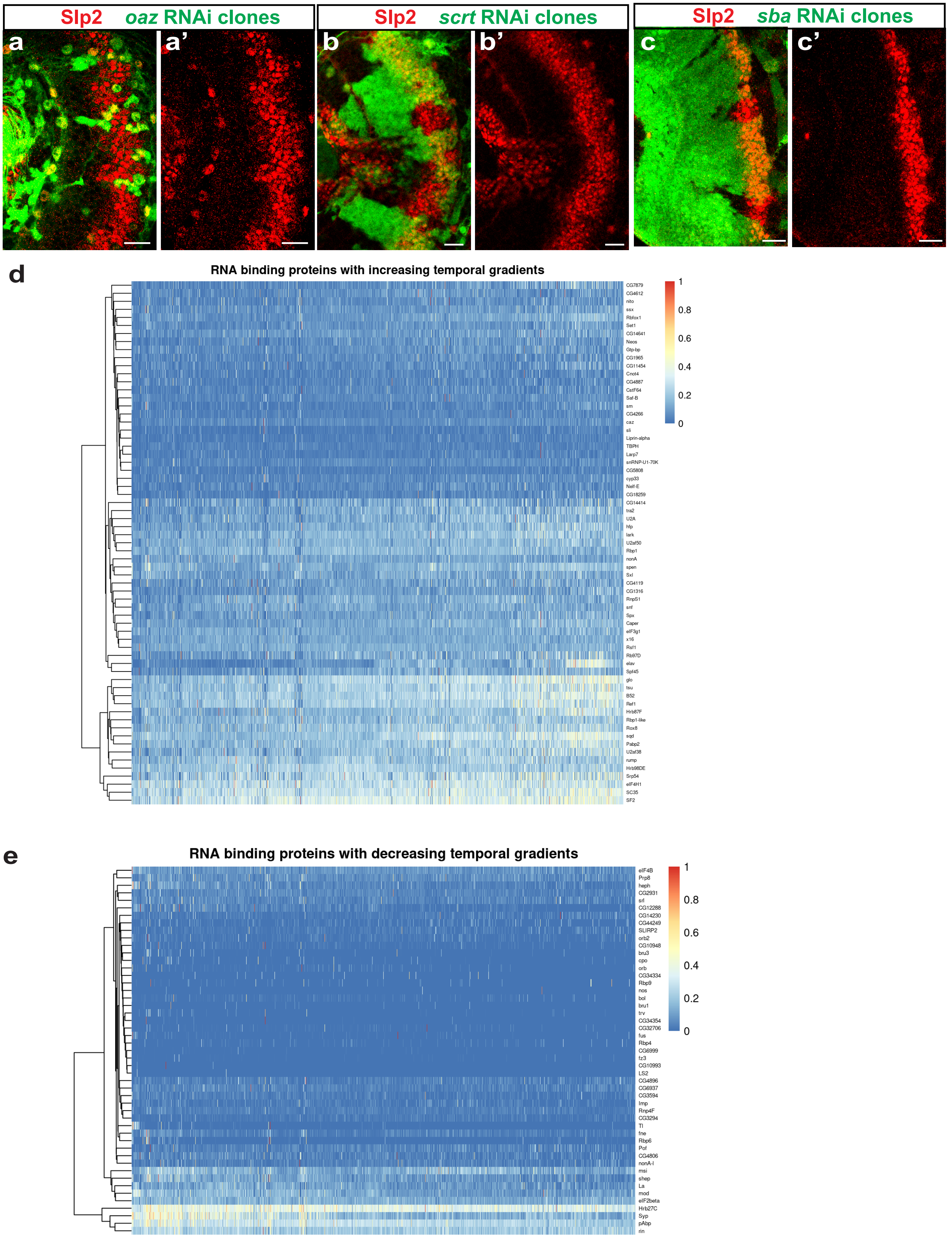

Supplementary Figure 6. Other temporally expressed transcription factors and RNA-binding proteins. (a-c') The expression of Slp2 (red) is not significantly affected in RNAi clones knocking down Oaz (a,a')(6 clones), Scrt (b,b')(5 clones), or Sba (c,c')(6 clones). (d, e) The expression patterns of a list of RNA binding proteins (compiled by flybase) were tested for Spearman correlation with the inferred pseudotime. (d) Heatmap of RNA-binding proteins that show an increasing temporal gradient with pseudotime. (e) Heatmap of RNA-binding proteins that show a decreasing temporal gradient with pseudotime. Scale bars: 20μm.

Supplementary Figure 7

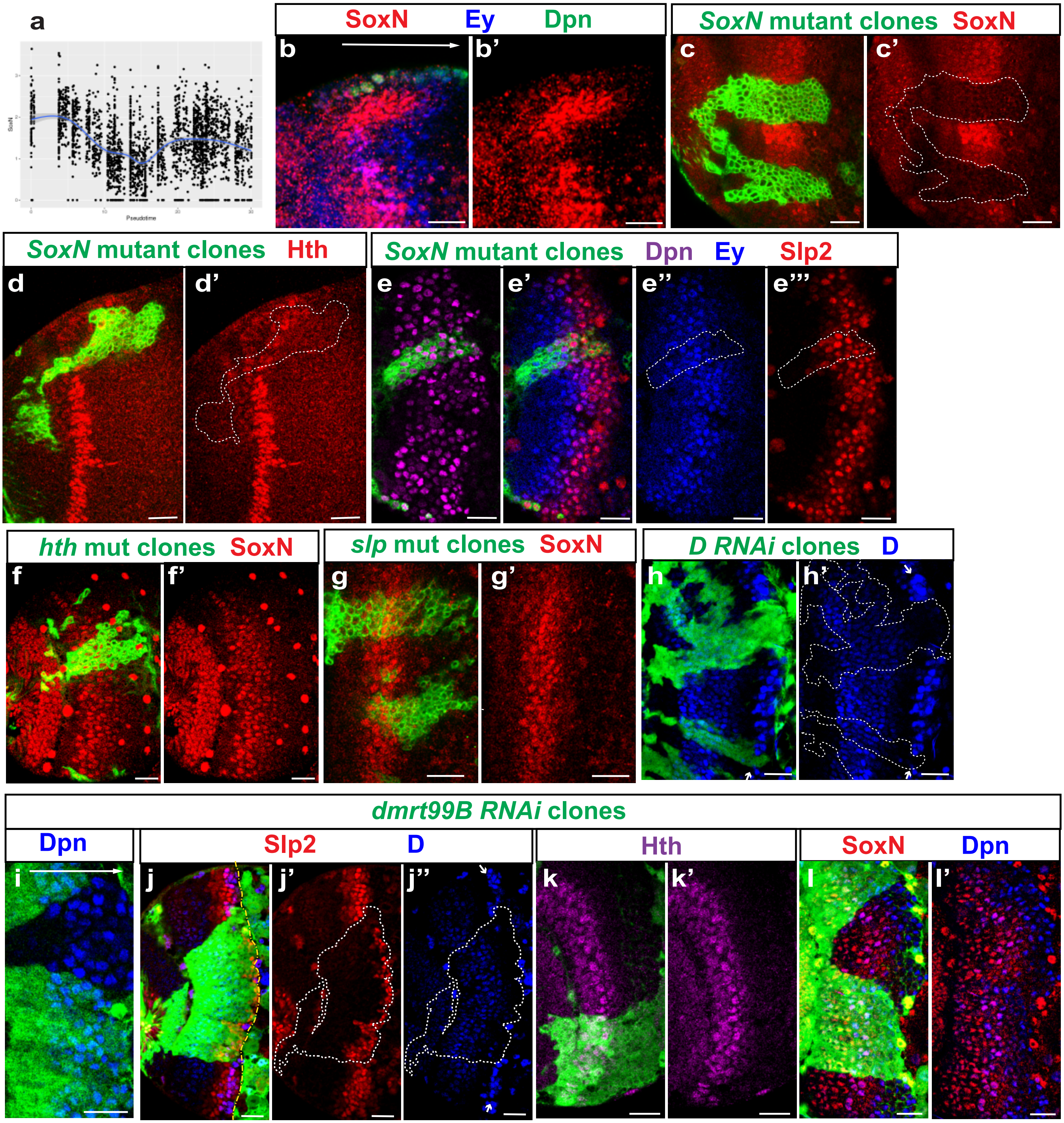

Supplementary Figure 7. Additional data for SoxN and Dmrt99B. (a) The expression pattern of SoxN along pseudotime showed two peaks. The shaded region indicates pointwise 95% confidence intervals. (b,b') SoxN (red) is expressed in progeny generated before the Ey (blue) expressing progeny. NBs are marked by Dpn (green). (c,c') In *SoxN*^{NC14} mutant clones marked by GFP (green), SoxN (red) is lost (6 out of 6 clones). (d,d') In *SoxN*^{NC14} mutant clones marked by GFP (green), Hth expression (red) is normal (5 out of 5 clones). (e-e''') In *SoxN*^{NC14} mutant clones marked by GFP (green), Ey (blue) and Slp2 (red) are still expressed in NBs marked by Dpn (magenta), but Slp2 expression is advanced (9 out of 9 clones). (f,f') In *hth* mutant clones marked by GFP (green), SoxN expression (red) is not affected (6 out of 6 clones). (g,g') In *slp* mutant clones marked by GFP (green), SoxN expression (red) is not affected (7 out of 7 clones). (h,h') The antibody for D (blue) has unspecific cross-reactivity to other proteins in early NBs (the weak staining that is not lost in *D-RNAi* clones), while the second stripe of strong staining (white arrows) is specific for D, as the staining is lost in *D-RNAi* clones (green)(in 5 out of 5 clones). (i) In *Dmrt99B-RNAi* clones marked by GFP (green), NB formation is not affected as indicated by Dpn staining (blue) (11 out of 11 clones). (j,j') In *Dmrt99B-RNAi* clones marked by GFP (green), Slp expression (red) is delayed, and D expression (strong blue staining indicated by white arrows) is lost (in 8 out of 8 clones). Note the weak blue staining in young NBs is cross-reactivity against other proteins. The white dashed lines in J'-J'' indicate clone margins, while the yellow dashed line with alternating short and long dashes in J indicates the boundary between the medulla and the central brain. (k,k') In *Dmrt99B-RNAi* clones marked by GFP (green), Hth expression (magenta) in the neuroblast layer is not affected (12 out of 12 clones). (l,l')) In *Dmrt99B-RNAi* clones marked by GFP (green), SoxN expression (red) is still expressed in the neuroblasts (marked by Dpn in blue) (in 10 out of 10 clones). Scale bars: 20µm.

Supplementary Figure 8

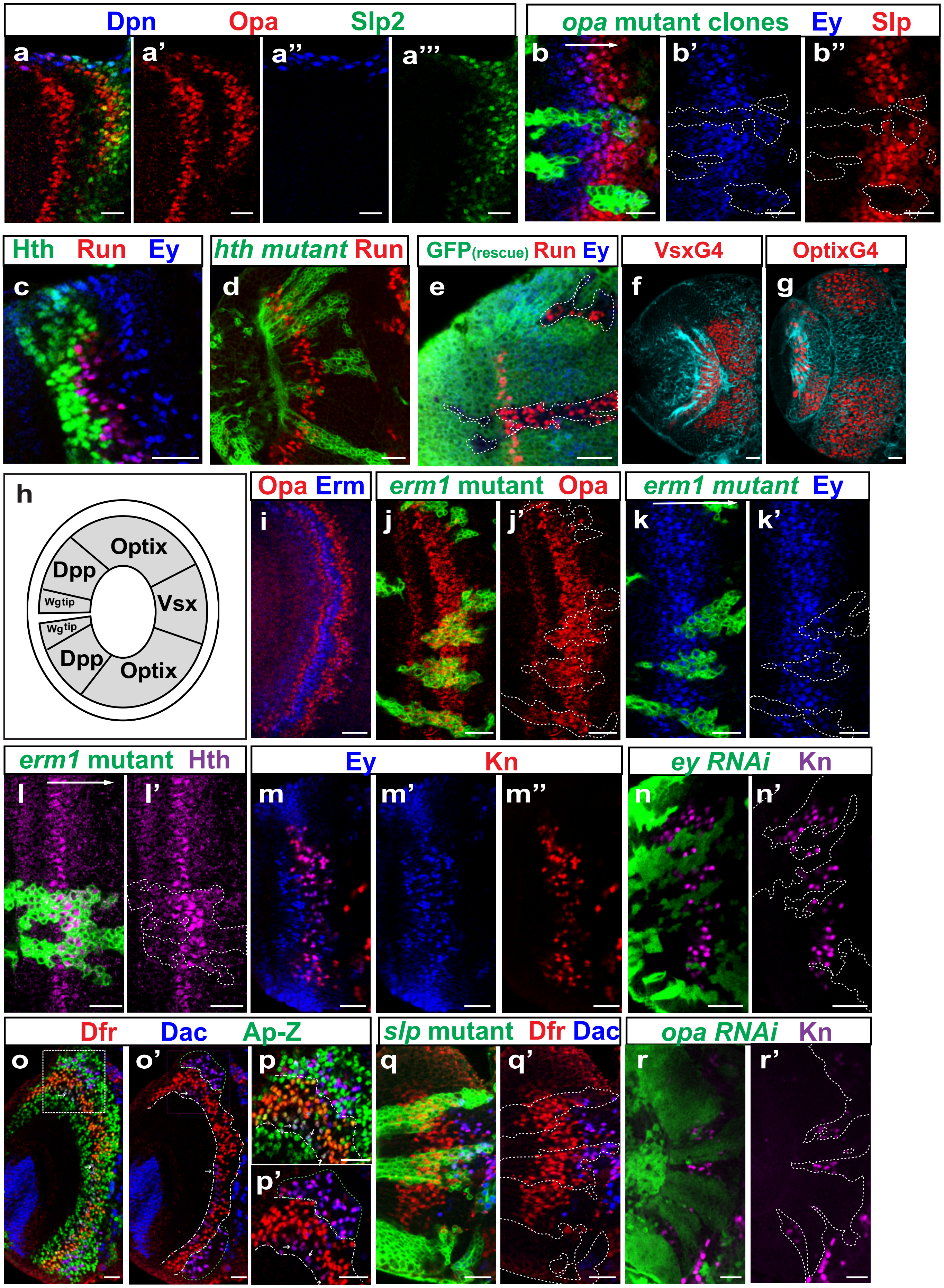

Supplementary Figure 8. **Additional data for Opa and Erm.** (a-a''') In a cross-sectional view, Opa (red) is expressed in two layers of progeny, but is lost as neurons mature. Slp2 expression is in green. The NB layer is marked by Dpn (blue). (b-b'') In opa^7 mutant clones (marked by GFP in green), the expression of Ey (blue) and Slp (red) is greatly delayed (in 14 out of 14 clones). (c) Runt (red) expressing neurons are generated between the Hth (green) neurons and the Ey (blue) stage neurons. Note: Runt neurons later turn on Ey expression, but they are not generated in the Ey stage. (d) In hth^{P2} mutant clones marked by GFP (green), Runt (red) expressing neurons are still generated (8 out of 8 clones). (e) In *ey* mutant clones marked by lack of GFP (green), Ey (blue) is lost, Runt (red) is expanded into later-born progeny (10 out of 10 clones). These negatively marked clones are generated in *ey* mutant background, and GFP positive cells contain the *eyBAC* rescue construct, while GFP negative cells are *ey* mutant (enclosed by white dashed lines). (f) vsxG4 (red) is expressed in the central region of the medulla (cOPC). (g) optixG4 (red) is expressed in ventral and dorsal main regions of the medulla (mOPC). (h) The schematic model of the compartmentalization of the medulla by marker genes. (i) Erm::V5 (blue) is expressed in a layer of progeny generated between the two layers of Opa (red) expressing progeny. (j,j') In erm^1 mutant clones marked by GFP (green) at a deeper progeny focal plane, the gap of Opa (red) expression in the progeny is also lost (in 13 out of 13 clones). (k,k') In erm^1 mutant clones marked by GFP (green) at the surface NB focal plane, Ey expression (blue) is still present but weaker (in 10 out of 10 clones). (l,l') In erm^1 mutant clones marked by GFP (green), Hth expression (magenta) is slightly expanded (in 5 out of 5 clones). (m-m'') Kn (red) is expressed in the Ey-expressing (blue) neurons generated by the Ey stage NBs. Note: A layer of earlier-born Ey expressing neurons (which are the Runt neurons that turn on Ey expression later), do not express Kn. (n,n') In *ey-RNAi* clones marked by GFP (green), Kn (magenta) neurons are lost (in 10 out of 10 clones). (o-p')The expression of Dfr (red), Dac (blue), and *ap*-LacZ (green) in neurons: a layer of earlier-born neurons express both Dfr and *ap*-LacZ (between the two white dashed lines in (o'-p'), and several clusters of later-born neurons express both Dfr and Dac but not *ap*-LacZ (enclosed in green dashed circles). White arrows point to cells expressing Dfr, *ap*-LacZ and a weak level of Dac within the first population. (p,p') A zoomed-in image of the outlined square in o. (q,q') In *slp* mutant clones marked by GFP (green), Dfr (red) and Dac (blue) expressing neurons are still generated (10 out of 12 clones). (r,r') In *opa-RNAi* clones marked by GFP (green), Kn (magenta) expression is lost (in 19 out of 20 clones). Scale bars: 20µm.

Supplementary Figure 9

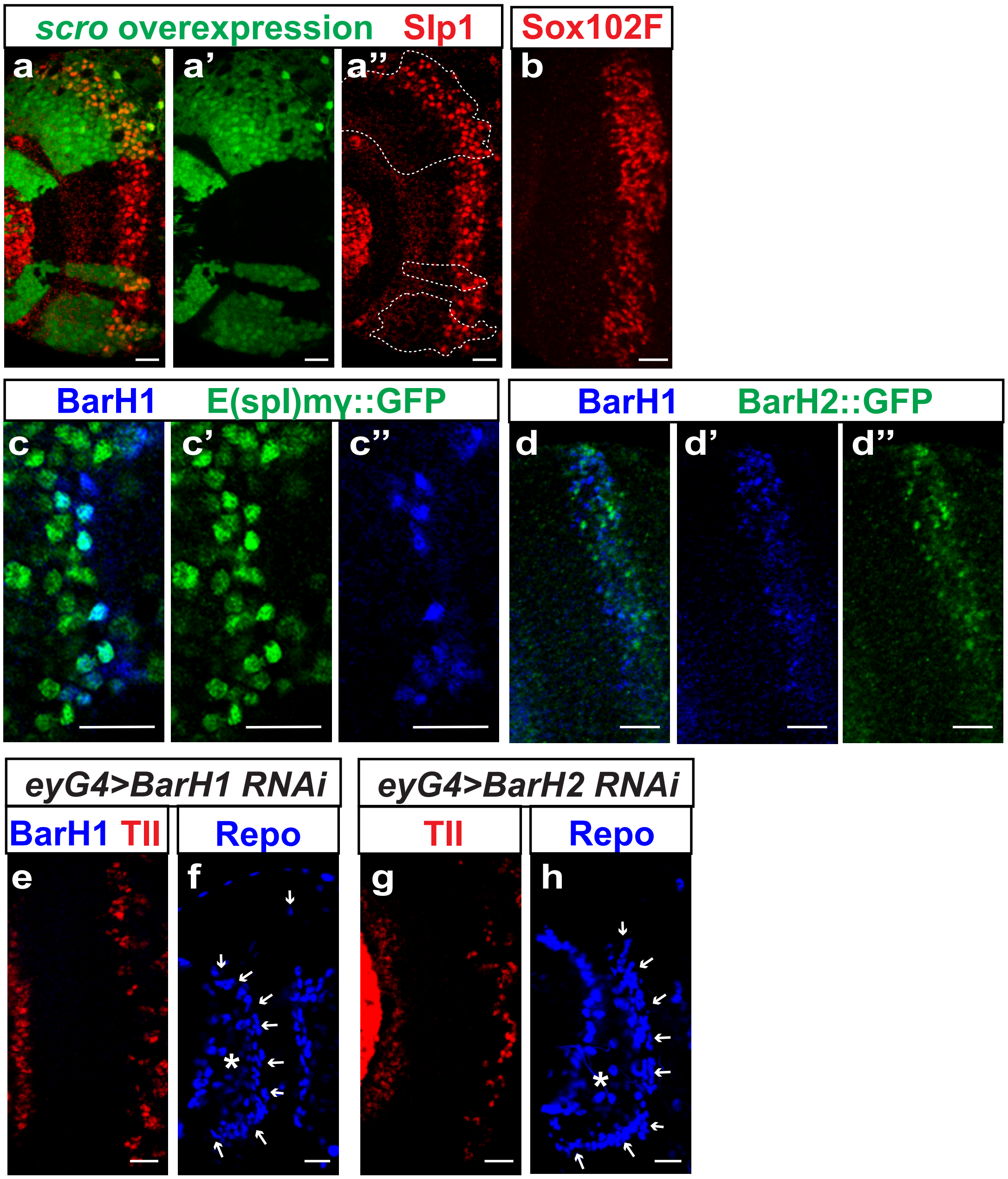

Supplementary Figure 9: Additional data for Scro and BarH.
(a) In clones marked by GFP (green) that have misexpression of Scro, Slp1 expression (red) is not affected (in 9 out of 9 clones). (b) The expression pattern of Sox102F (red) in neurons. (c-c'') The expression of BarH1 (blue) in old medulla NBs marked by E(spl)mγ::GFP (green). E(spl)mγ::GFP is expressed in all NBs. (d-d'') The expression of BarH1 (blue) and BarH2::GFP(green) in neurons in a cross-sectional view. (e) At the surface NB focal plane, Tll (red) expression in NBs is not affected when *BarH1* RNAi is driven by *eyGal4* (in 4 out of 4 brains). (f) At a deep progeny focal plane, glia marked by Repo (blue) are not affected (in 4 out of 4 brains) when *BarH1* RNAi is driven by *eyGal4*. The asterisk indicates the center of the neuropil, and the arrows are pointing at the glia. (g) At the surface NB focal plane, Tll (red) is not affected in NBs when *BarH2* RNAi is driven by *eyGal4* (in 3 out of 3 brains). (h) At a deep progeny focal plane, glia marked by Repo (blue) are not affected when *BarH2* RNAi is driven by *eyGal4* (in 3 out of 3 brains). The asterisk indicates the center of the neuropil, and the arrows are pointing at the glia. Scale bars: 20µm.

Supplementary Figure 10

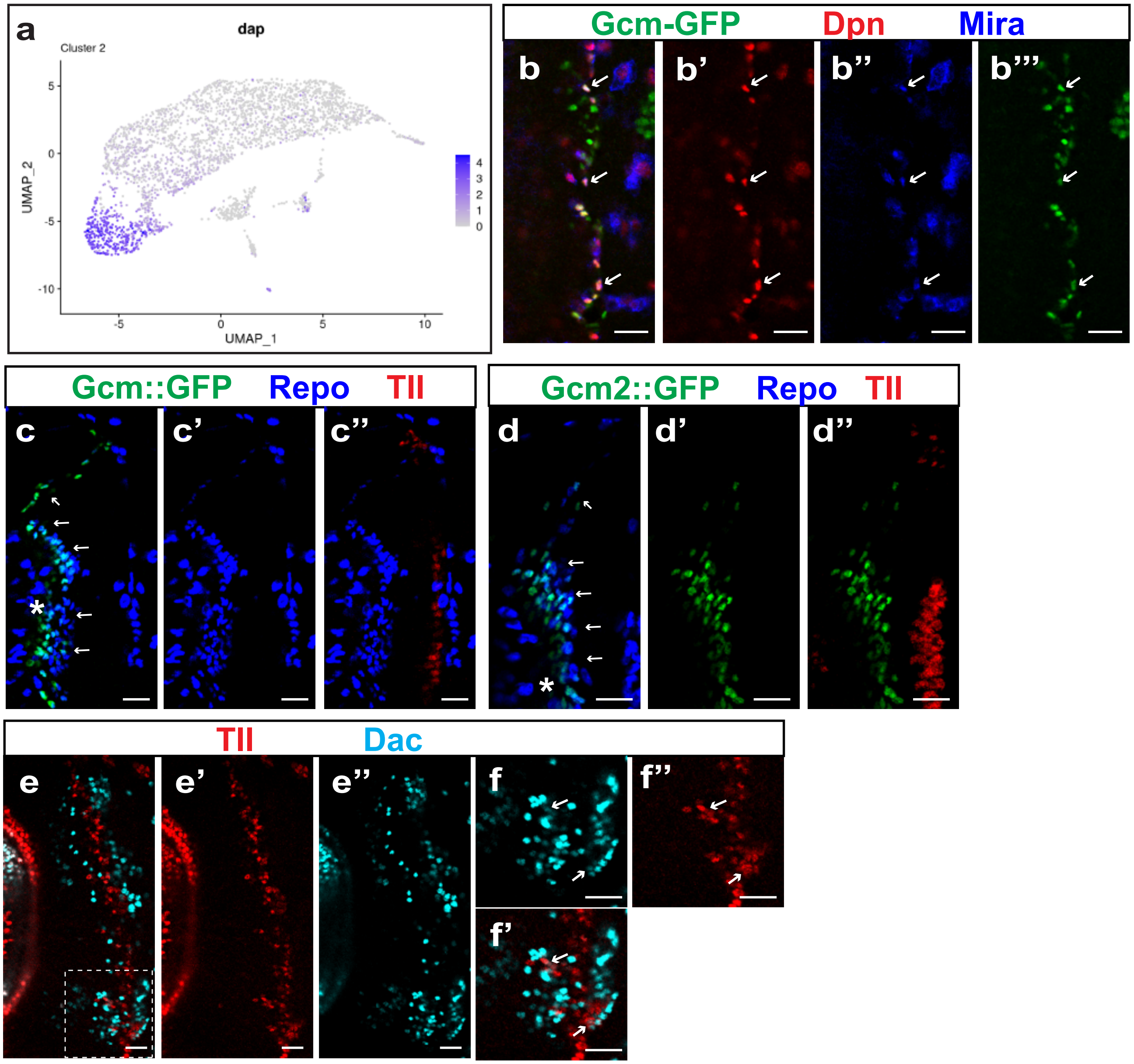

Supplementary Figure 10: Additional data for Gcm.

(a) The mRNA expression pattern of Dap from the scRNA-seq data visualized on UMAP Plot. (b-b''') Gcm-GFP (green), Dpn (red) and Mira (blue) are co-expressed in the oldest NBs. The arrows are pointing at some of such NBs expressing all three markers. (c-c'') The expression of Tll (red), Gcm::GFP (green) and Repo (blue) in a cross-sectional view. The asterisk indicates the center of the neuropil, and arrows point to mng. (d-d'') The expression of Tll (red), Gcm2::GFP (green) and Repo (blue) in a cross-sectional view. The asterisk indicates the center of the neuropil, and arrows point to mng. (e-f'') At a relatively superficial progeny focal plane close to the surface NBs, a few Tll (red) and Dac (blue) double positive neurons are present in the wild-type brain. (f-f') A zoomed-in image of the outlined region in panel e. Arrows point to neurons expressing Tll and a weak level of Dac. Tll^{+} Dac^{+} neurons are very rare in wild type brains, and Dac expression in those neurons is weak. One possibility is that Dac level is only increased in Tll stage neurons after Tll is lost. While in *gcm* mutant or RNAi clones, the abnormal maintenance of Tll expression in neuroblasts may also lead to abnormal maintenance of Tll expression in neurons. This together with the increased production of Tll stage neurons enable us to see a lot of $Tll^{+}Dac^{+}$ neurons. Another possibility is that in wild type brains strong Dac is not normally observed in Tll stage neurons, because the transient expression of Tll in neurons is unable to effectively activate strong Dac expression. While with loss of Gcm, strong Tll expression in neurons is maintained longer to ectopically activate strong Dac. Scale bars: 20µm.

Supplementary Figure 11

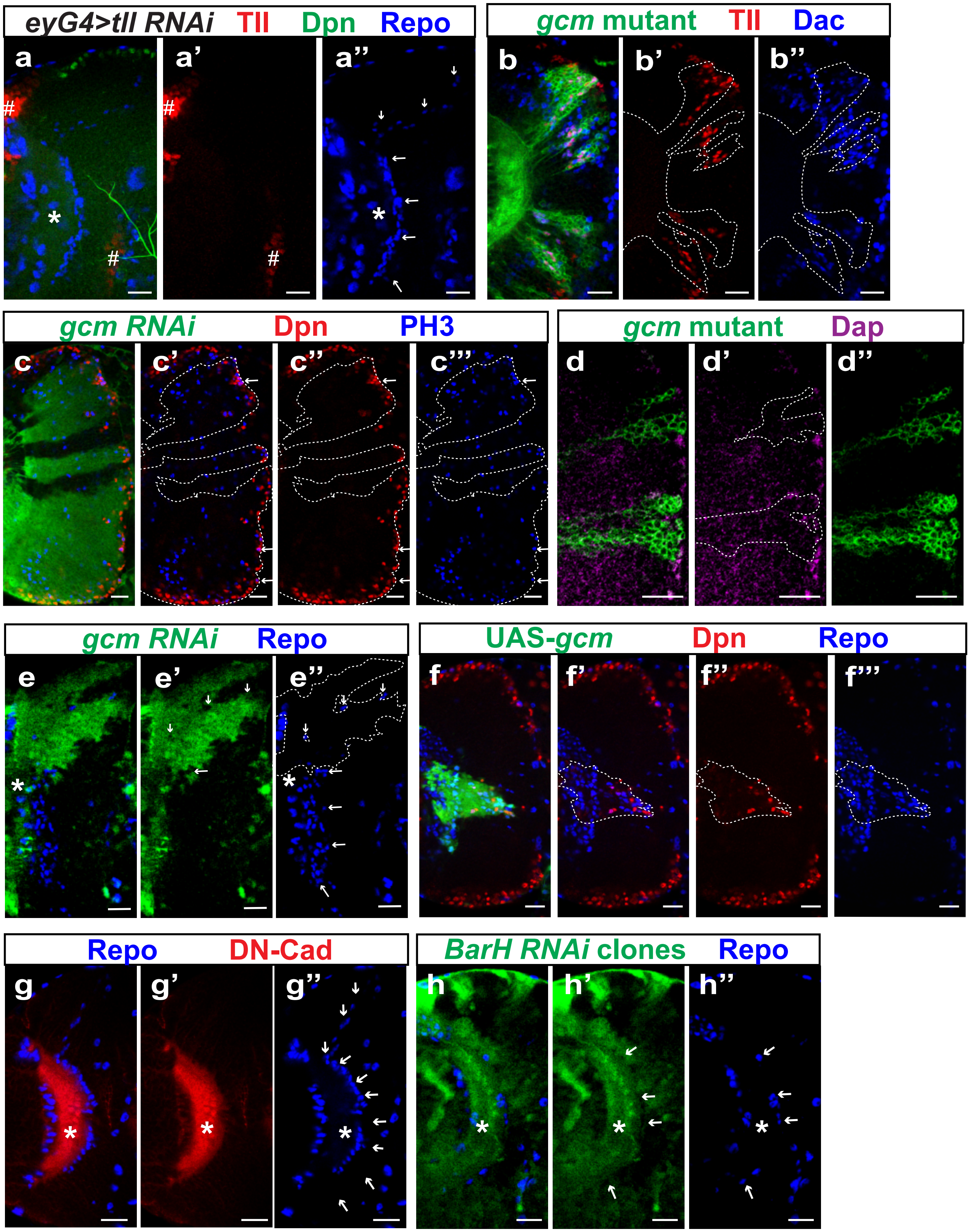

Supplementary Figure 11: Additional data for the function of Gcm.

(a-a'') When *tll* RNAi (*tll*-miRNA) is driven by *eyGal4*, Tll (red) is lost in NBs marked by Dpn (green), while glia marked by Repo (blue) are still aligned continuously around the medulla neuropil (in 3 out of 3 brains). The asterisk indicates the center of the neuropil, and arrows are pointing at the glia. The "#" symbol indicates Tll expression in the NE of OPC and IPC is not affected because eyGal4 is not expressed there. (b-b'') In *gcm* mutant clones (green), Tll (red) and Dac (blue) double positive cells are increased (in 15 out of 15 clones). (c-c''') In *gcm-RNAi* clones, some of the oldest NBs shows PH3 staining (in 8 out of 10 clones). (d-d'') In *gcm* mutant clones (green), Dap (magenta) is lost (in 6 out of 6 brains). (e-e'') In *gcm-RNAi* clones (marked by GFP in green), glia marked by Repo (blue) are mostly lost (in 12 out of 12 clones). The asterisk indicates the center of the neuropil, and the arrows are pointing at the glia. Note the glia don't have GFP expression. (f-f''') In *gcm* misexpression clones (green), NBs marked by Dpn (red) are surrounded by ectopic glia marked by Repo (blue) (in 13 out of 13 clones). (g-g'') In wild-type brains, mng marked by Repo (blue) migrate towards the neuropil marked by DN-Cad (red), and are aligned continuously around the neuropil surface. The asterisk indicates the center of the neuropil, and the arrows are pointing at the glia. (h-h'') In *BarH1* and *BarH2* double RNAi clones marked by GFP (green), mng marked by Repo (blue) is greatly lost (in 6 out of 6 clones). Arrows point to the remaining glia that lie outside of the clones. Scale bars: 20µm.

Supplementary Figure 12

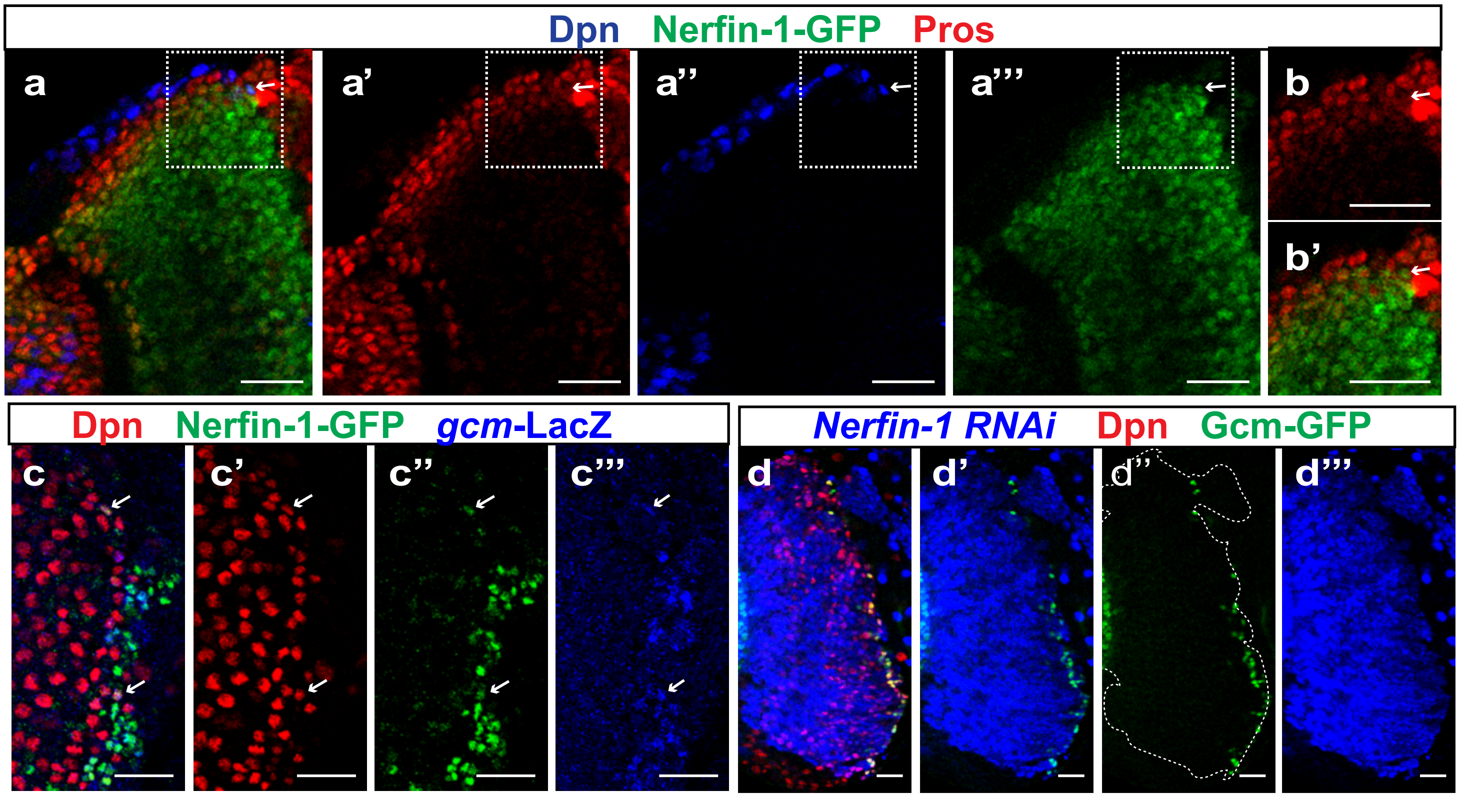

Supplementary Figure 12: Additional data for Nerfin-1.

(a-b') The expression pattern of Dpn (blue), Nerfin-1-GFP (green) and Pros (red) in a cross-sectional view. The arrow is pointing at a NB that express both Nerfin-1-GFP and weak Pros. (b-b') A zoomed-in image of the outlined region in panel A. (c-c''') The expression pattern of Dpn (red), Nerfin-1-GFP (green) and Gcm-lacZ (blue) at the surface layer. The arrows are pointing at NBs that express both Nerfin-1-GFP and β-galactosidase. (d-d''') In *Nerfin-1-RNAi* clones marked by β-gal in blue, the expression of Gcm-GFP (green) is still present (in 15 out of 25 clones). Scale bars: 20μm.

Supplementary Figure 13

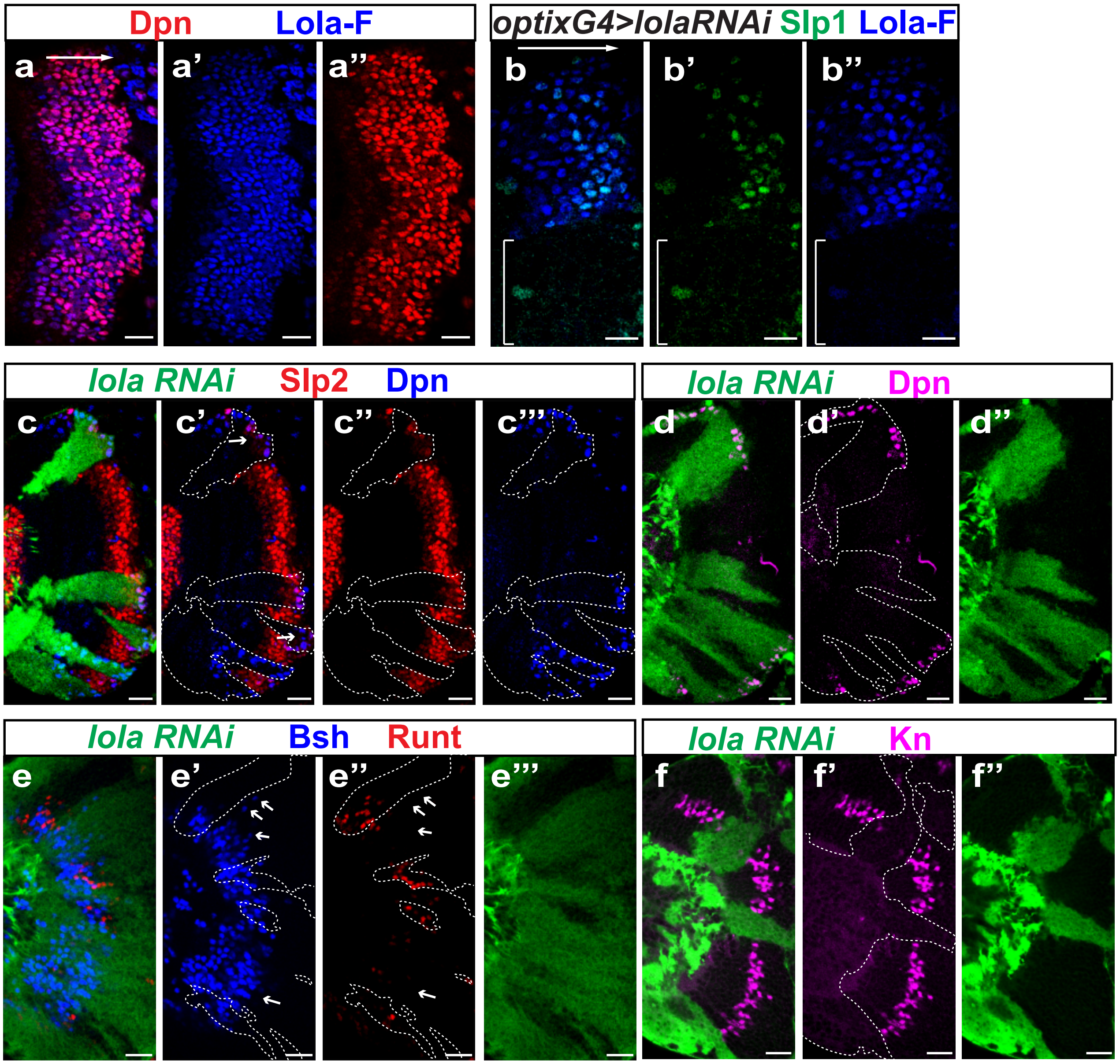

Supplementary Figure 13: Additional data for Lola. (a-a'') The expression of Dpn (red) and Lola-F (blue) at a surface focal plane. (b-b'') When *lola* RNAi (BDSC35721) is driven by *optixGal4*, the expression of Lola-F (blue) is lost and the expression of Slp1 (green) is greatly delayed (in 4 out of 4 brains) in mOPC indicated by a white bracket. (c-c''') In *lola-RNAi* (VDRC 101925) clones marked by GFP (green), the delayed activation of Slp2 (red) can be observed at a slightly deeper focal plane where some oldest NBs can be seen (white arrows) (in 24 out of 24 clones). (d-d'') In *lola-RNAi* (VDRC 101925) clones marked by GFP (green), at a deep progeny focal plane, some ectopic NBs (magenta) can be observed at the medial edge of the clones (in 60 out of 60 clones). (e-e''') In *lola-RNAi* (VDRC 101925) clones marked by GFP (green), Runt neurons are lost, while Bsh neurons are slightly increased (white arrows)(in 8 out of 8 clones). (f-f'') In *lola-RNAi* (VDRC 101925) clones, Kn neurons are mostly lost (in 12 out of 12 clones). Scale bars: 20µm.

Supplementary Table 1. Known *Drosophila* cell cycle genes from Tinyatlas at Github (source: https://github.com/hbc/tinyatlas/blob/master/cell_cycle/Drosophila_melanogaster.csv). This table includes known *Drosophila* cell cycle genes and the corresponding phase, and were used to estimate the cell cycle phase of each cell in our scRNA-seq data.

Supplementary Table 2. The top 200 genes showing decreasing gradients of expression and the enriched GOTERM analysis. Genes with temporally patterned expression gradients were identified as those whose expression levels showed significant non-zero Spearman correlation with the inferred pseudotime at an FDR cutoff of 0.05. The testing is two-sided. These genes were sorted according to the "corr" column (Spearman correlation), and the top 200 genes with the highest negative values (sheet1) were analyzed for enriched Go terms for Biological Processes "GOTERM_BP_DIRECT" using the "Functional Annotation Chart" at the DAVID Bioinformatics Resources 6.8 website (https://david.ncifcrf.gov/home.jsp). The whole output from the Functional Annotation Chart is in sheet2. DAVID program uses modified Fisher's exact test to calculate P values for the significance of enrichment, and multiple testing correction techniques (Bonferroni, Benjamini and FDR) are provided. Those with FDR<0.15 are highlighted.

Supplementary Table 3. The top 200 genes showing increasing gradients of expression and the enriched GOTERM analysis. Genes with temporally patterned expression gradients were sorted according to the "corr" column (Spearman correlation), and the top 200 genes with the highest positive values (sheet 1) were analyzed for enriched Go terms for Biological Processes "GOTERM_BP_DIRECT" using the "Functional Annotation Chart" at the DAVID Bioinformatics Resources 6.8 website (https://david.ncifcrf.gov/home.jsp). The whole output from the Functional Annotation Chart is in sheet2. DAVID program uses modified Fisher's exact test to calculate P values for the significance of enrichment, and multiple testing correction techniques (Bonferroni, Benjamini and FDR) are provided. Those with FDR<0.15 are highlighted.

Supplementary Table 4. List of reagents used in this study with sources and Identifier numbers.

Supplementary Table 5. List of antibodies and reporter lines used to examine the protein expression pattern of differentially expressed transcription factors.

FACS Gating Information

We used SSC-A/FSC-A to exclude debris, and then single cells were selected based on FSC-A/FSC-W. Live cells were selected based on low DAPI staining (Pacific Blue-A). Control cells without GFP or RFP fluorescence were used to set the threshold on FITC-A(GFP) and PE-A(RFP) so that 0% of control cells are GFP+RFP+. For the sample, selected GFP+RFP+ cells were 1 order of magnitude more fluorescent than other cells on FITC-A, and 2-3 orders of magnitude more fluorescent than other cells on PE-A.

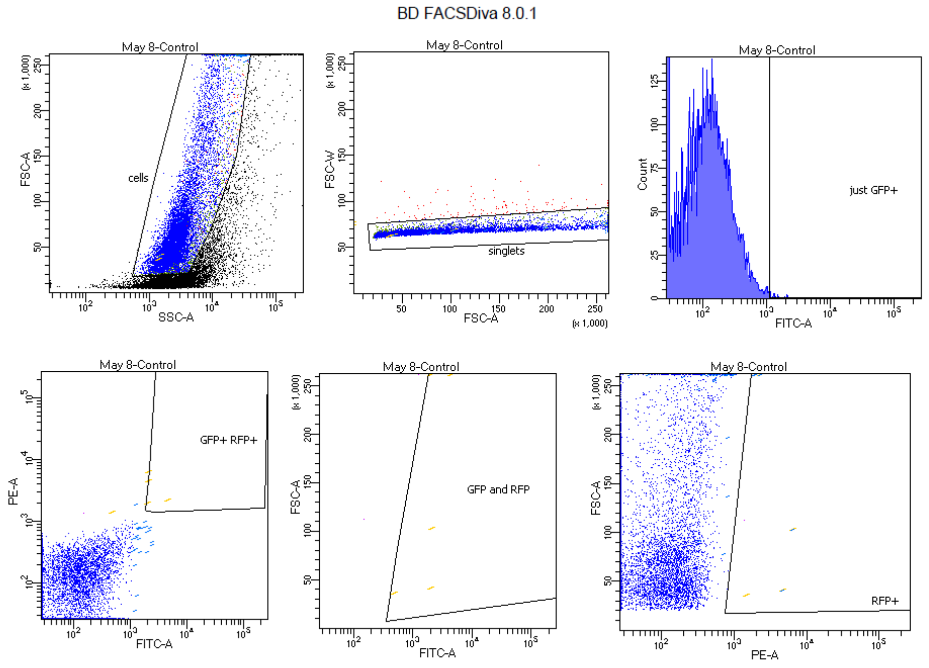

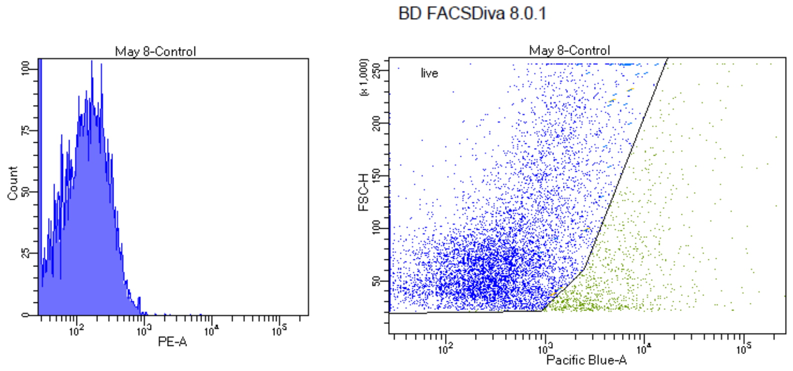

Tube: Control

Population	#Events	%Parent	%Total
All Events	18,052	####	100.0
cells	10,396	57.6	57.6
singlets	10,286	98.9	57.0
live	9,282	90.2	51.4
RFP+	7	0.1	0.0
GFP and RFP	5	71.4	0.0
GFP+ RFP+	3	0.0	0.0
just GFP+	28	0.3	0.2

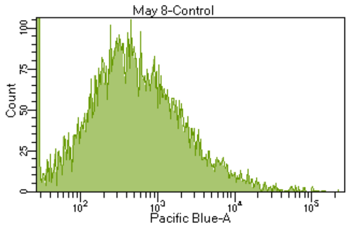

BD FACSDiva 8.0.1

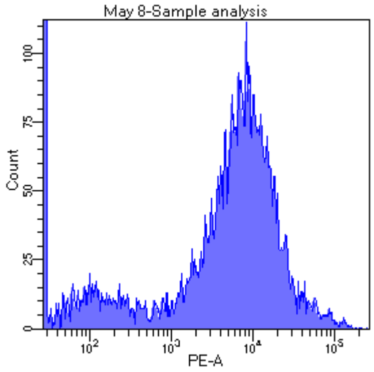

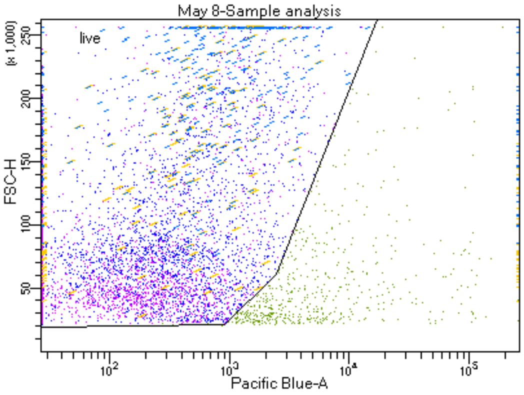

Tube: Sample analysis

Population	#Events	%Parent	%Total
All Events	15,453	####	100.0
cells	10,766	69.7	69.7
singlets	10,647	98.9	68.9
live	10,107	94.9	65.4
RFP+	4,807	47.6	31.1
GFP and RFP	165	3.4	1.1
GFP+ RFP+	14	0.1	0.1
just GFP+	260	2.6	1.7

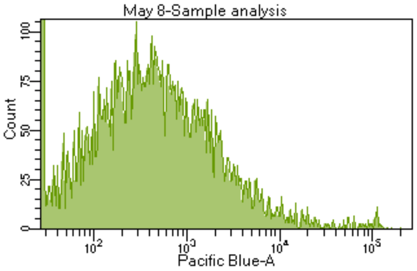

BD FACSDiva 8.0.1

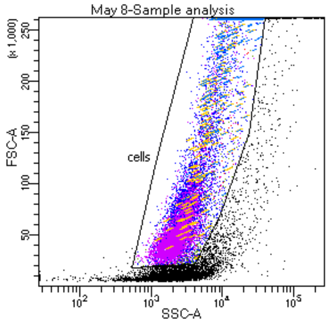

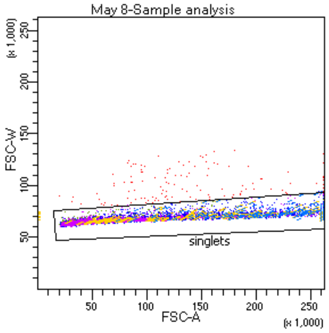

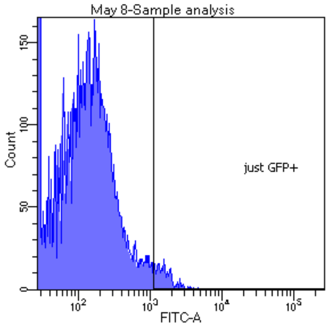

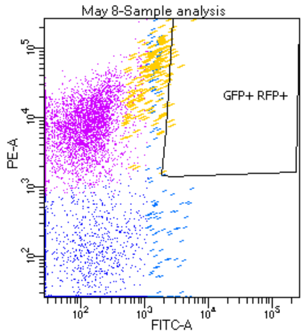

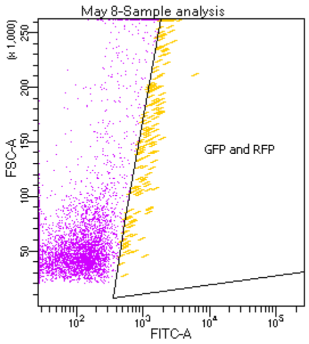

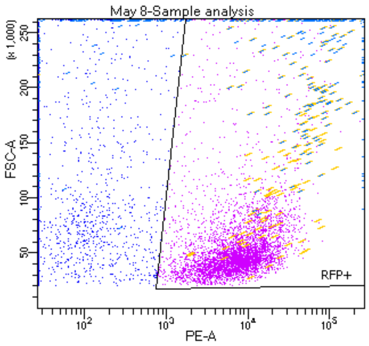

BD FACSDiva 8.0.1

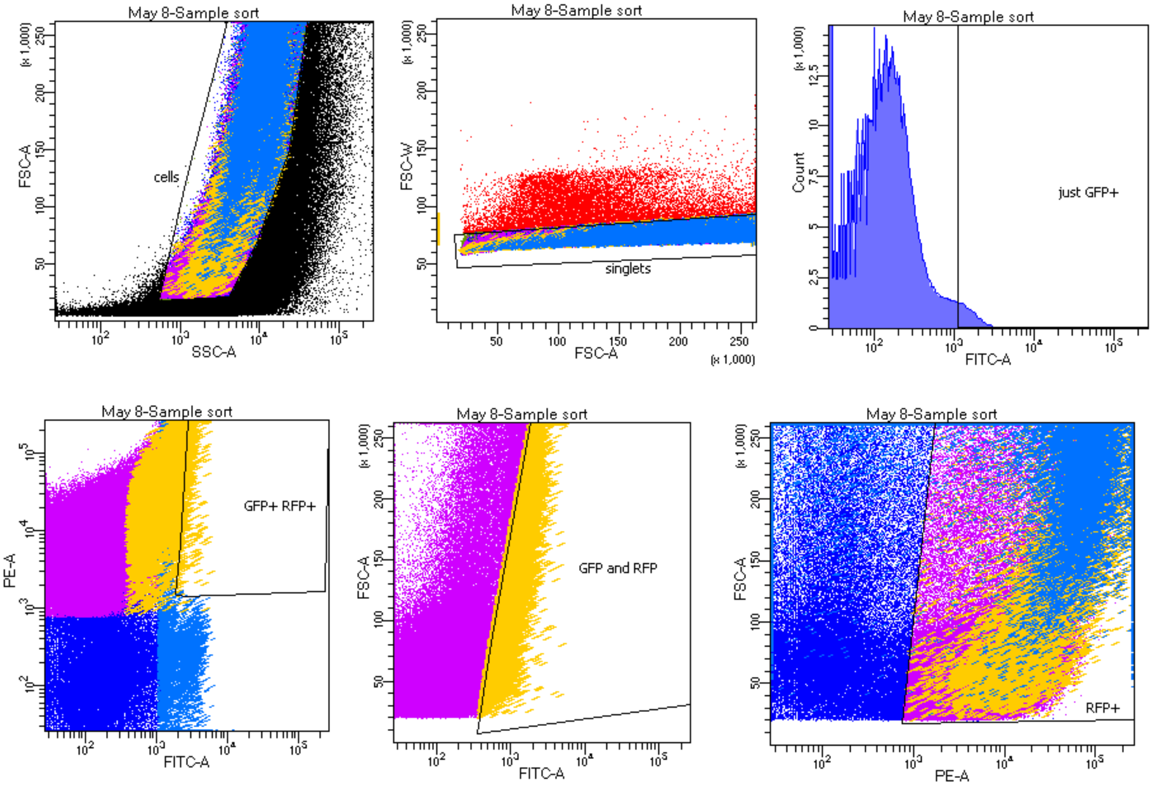

BD FACSDiva 8.0.1

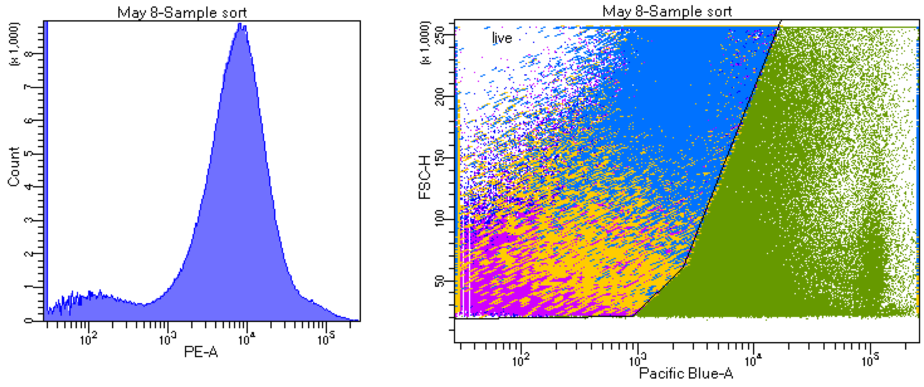

Tube: Sample sort

Population	#Events	%Parent	%Total
All Events	1,701,467	####	100.0
cells	1,166,912	68.6	68.6
singlets	1,152,998	98.8	67.8
live	1,002,323	86.9	58.9
RFP+	498,282	49.7	29.3
GFP and RFP	13,149	2.6	0.8
GFP+ RFP+	574	0.1	0.0
just GFP+	21,172	2.1	1.2

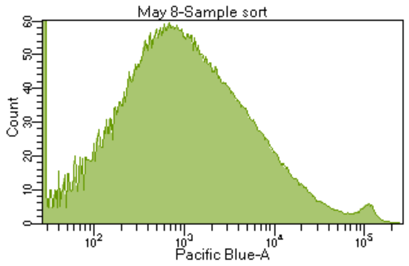

Supplement: Supplementary file 1 — Supplementary Information [file 41467_2022_28915_MOESM1_ESM.pdf]
