# Supplementary material for: A comprehensive temporal patterning gene network in Drosophila medulla neuroblasts revealed by single-cell RNA sequencing
Source: Nat Commun. 2022 Mar 10;13:1247. doi: 10.1038/s41467-022-28915-3 (PMC8913700; doi:10.1038/s41467-022-28915-3)
Supplement: Supplementary file 8 — Reporting Summary [file 41467_2022_28915_MOESM8_ESM.pdf]

nature portfolio

Corresponding author(s): Xin Li

Last updated by author(s): Jan 11, 2022

Reporting Summary

Nature Portfolio wishes to improve the reproducibility of the work that we publish. This form provides structure for consistency and transparency in reporting. For further information on Nature Portfolio policies, see our Editorial Policies and the Editorial Policy Checklist.

Statistics

For all statistical analyses, confirm that the following items are present in the figure legend, table legend, main text, or Methods section.

n/a	Confirmed	
☐	☒	The exact sample size (*n*) for each experimental group/condition, given as a discrete number and unit of measurement
☐	☒	A statement on whether measurements were taken from distinct samples or whether the same sample was measured repeatedly
☐	☒	The statistical test(s) used AND whether they are one- or two-sided<br>*Only common tests should be described solely by name; describe more complex techniques in the Methods section.*
☒	☐	A description of all covariates tested
☒	☐	A description of any assumptions or corrections, such as tests of normality and adjustment for multiple comparisons
☐	☒	A full description of the statistical parameters including central tendency (e.g. means) or other basic estimates (e.g. regression coefficient) AND variation (e.g. standard deviation) or associated estimates of uncertainty (e.g. confidence intervals)
☐	☒	For null hypothesis testing, the test statistic (e.g. *F*, *t*, *r*) with confidence intervals, effect sizes, degrees of freedom and *P* value noted<br>*Give P values as exact values whenever suitable.*
☒	☐	For Bayesian analysis, information on the choice of priors and Markov chain Monte Carlo settings
☒	☐	For hierarchical and complex designs, identification of the appropriate level for tests and full reporting of outcomes
☒	☐	Estimates of effect sizes (e.g. Cohen's *d*, Pearson's *r*), indicating how they were calculated

Our web collection on statistics for biologists contains articles on many of the points above.

Software and code

Policy information about availability of computer code

Data collection	Zen Black 2012 SP5 confocal software, Release version 14.0.0.0.
Data analysis	R (version 4.0.3)<br>Seurat (version 3.2.3)<br>Monocle3 (version 0.2.3.0) (Batchelor algorithm is part of the Monocle3)<br>David 6.8<br>Cell Ranger (version3.0.1, and 3.1.0)<br>Customized codes available at 10.5281/zenodo.5813627<br>Adobe Photoshop CC 2017<br>Adobe Illustrator CC 2017

For manuscripts utilizing custom algorithms or software that are central to the research but not yet described in published literature, software must be made available to editors and reviewers. We strongly encourage code deposition in a community repository (e.g. GitHub). See the Nature Portfolio guidelines for submitting code & software for further information.

Data

Policy information about availability of data

All manuscripts must include a data availability statement. This statement should provide the following information, where applicable:

- Accession codes, unique identifiers, or web links for publicly available datasets
- A description of any restrictions on data availability
- For clinical datasets or third party data, please ensure that the statement adheres to our policy

The raw and processed scRNA-seq data generated in this study have been deposited in the NCBI's Gene Expression Omnibus database (GEO) under accession code GSE168553 [https://www.ncbi.nlm.nih.gov/geo/query/acc.cgi?acc=GSM5145863]. Ensembl's BDGP6.22 is available at [http://sep2019.archive.ensembl.org/Drosophila_melanogaster/Info/Annotation].

Field-specific reporting

Please select the one below that is the best fit for your research. If you are not sure, read the appropriate sections before making your selection.

☒ Life sciences ☐ Behavioural & social sciences ☐ Ecological, evolutionary & environmental sciences

For a reference copy of the document with all sections, see nature.com/documents/nr-reporting-summary-flat.pdf

Life sciences study design

All studies must disclose on these points even when the disclosure is negative.

Sample size	No statistical approach was used to predetermine sample size. Samples sizes were determined following standards in the field and our previous experience (Li, X. et al. 2013)(Erclik, T. et al. 2017)
Data exclusions	No data exclusion.
Replication	All experiments were done independently at least twice, and each time there were 6-15 biological replicates. Conclusions are only drawn when consistent results were found.
Randomization	Not relevant, because all animals (Drosophila) have the same genetic background and age.
Blinding	The scRNA-seq was performed in wild type animals, therefore blinding across different samples is not applicable. For most immunostaining experiments, we are comparing gene expression in GFP marked mutant clones with regions outside of the clones. Mutant clones are marked by GFP, so blinding is not practical for our experiments.

Reporting for specific materials, systems and methods

We require information from authors about some types of materials, experimental systems and methods used in many studies. Here, indicate whether each material, system or method listed is relevant to your study. If you are not sure if a list item applies to your research, read the appropriate section before selecting a response.

Materials & experimental systems

n/a	Involved in the study	
☐	☒	Antibodies
☒	☐	Eukaryotic cell lines
☒	☐	Palaeontology and archaeology
☐	☒	Animals and other organisms
☒	☐	Human research participants
☒	☐	Clinical data
☒	☐	Dual use research of concern

Methods

n/a	Involved in the study	
☒	☐	ChIP-seq
☐	☒	Flow cytometry
☒	☐	MRI-based neuroimaging

Antibodies

Antibodies used

Antibodies / Source / IDENTIFIER/ CLONE # /LOT # /DILUTION
Rabbit anti SoxN, Steven Russell (Ferrero, Fischer and Russell, 2014) N/A N/A N/A 1:100
Rabbit anti-Hth, Richard Mann N/A N/A N/A 1:500
Guinea-pig anti-Run, Claude Desplan N/A N/A N/A 1:500
Rabbit anti-Bsh, Claude Desplan N/A N/A N/A 1:500
Rabbit anti-Slp1, Claude Desplan N/A N/A N/A 1:500

Guinea-pig anti-Slp2, Claude Desplan N/A N/A N/A 1:500
Rabbit anti-D, Claude Desplan N/A N/A N/A 1:500
Guinea-pig anti-Tll, Claude Desplan N/A N/A N/A 1:500
Rabbit anti-Sox102F, Claude Desplan N/A N/A N/A 1:500
Rabbit anti-D, John R. Nambu (Ma et al., 1998) N/A N/A N/A 1:1000
Rat anti-Dfr, Makoto Sato (Hasegawa et al., 2011) N/A N/A N/A 1:200
Guinea-pig anti-Kn, Adrian Moore (Jinushi-Nakao et al., 2007) N/A N/A N/A 1:500
Guinea-pig anti-Dpn, Chris Doe N/A N/A N/A 1:500
Rabbit anti-Opa, J. Peter Gergen (Koromila et al., 2020) N/A N/A N/A 1:100
Rat anti-BarH1, Tiffany Cook (Charlton-Perkins et al., 2011) N/A N/A N/A 1:200
sheep anti-GFP, AbD Serotec 4745-1051 N/A 1113 1:500
Chick anti-beta Galactosidase, Abcam ab9361 N/A GR3360178-4 1:1000
Rat anti-Histone H3 (phospho S28) antibody, Abcam ab10543 HTA28 GR3208319-3 1:500
rabbit anti-RFP, Abcam ab62341 N/A GR257333-1 1:1000
Mouse anti V5-Tag:DyLight®550, Bio-Rad MCA1360D550GA SV5-Pk1 1607 1:200
Rat anti-Deadpan, Abcam ab195173 11D1BC7 GR3352273-7 1:200
mouse anti-eyeless, DSHB anti-eyeless N/A 12/8/2016 1:10
mouse anti-Pros, DSHB MR1A N/A 7/3/2014 1:10
mouse anti-Repo, DSHB 8D12 anti-Repo N/A 12/4/2014 1:50
mouse anti Cut, DSHB 2B10 N/A 10/18/2018 1:10
mouse anti-Dac, DSHB mAbdac2-3 N/A 8/28/2014 1:20
mouse anti-Dap, DSHB NP1 N/A 9/17/2015 1:5
mouse anti-Lola-F (Lola zf5), DSHB 7F1-1D5 N/A 11/17/2016 1:20
Cy5 AffiniPure Donkey Anti-RatIgG, Jackson ImmunoResearch Laboratories Inc. 712-175-153 N/A 115494 1:500
Cy3 AffiniPure Donkey Anti-RatIgG, Jackson ImmunoResearch Laboratories Inc. 712-165-153 N/A 120429 1:500
Cy3 AffiniPure Donkey Anti-Guinea Pig IgG, Jackson ImmunoResearch Laboratories Inc. 706-165-148 N/A 117878 1:500
Alexa Fluor® 647 AffiniPureDonkey Anti-Guinea Pig, Jackson ImmunoResearch Laboratories Inc. 706-605-148 N/A 117997 1:500
Alexa Fluor® 488 AffiniPureDonkey Anti-Guinea Pig IgG, Jackson ImmunoResearch Laboratories Inc. 706-545-148 N/A 118980 1:500
Alexa Fluor® 647 AffiniPureDonkey Anti-Goat IgG, Jackson ImmunoResearch Laboratories Inc. 705-605-147 N/A 116978 1:500
DyLight 405 AffiniPure DonkeyAnti-Mouse IgG Jackson ImmunoResearch Laboratories Inc. 715-475-151 N/A 118326 1:100
Alexa Fluor® 647 AffiniPureDonkey Anti-Rabbit IgG, Jackson ImmunoResearch Laboratories Inc. 711-605-152 N/A 118217 1:500
Cy5 AffiniPure Donkey Anti-Mouse IgG, Jackson ImmunoResearch Laboratories Inc. 715-175-151 N/A 118562 1:500
Alexa Fluor® 647 AffiniPureDonkey Anti-Chicken IgY (IgG), Jackson ImmunoResearch Laboratories Inc. 703-605-155 N/A 114163 1:500
Alexa Fluor® 488 AffiniPureDonkey Anti-Mouse IgG, Jackson ImmunoResearch Laboratories Inc. 715-545-151 N/A 119921 1:500
Alexa Fluor® 488 AffiniPureDonkey Anti-Sheep, Jackson ImmunoResearch Laboratories Inc. 713-545-147 N/A 116551 1:500
DyLight 405 AffiniPure DonkeyAnti-Rat IgG, Jackson ImmunoResearch Laboratories Inc. 712-475-153 N/A 117198 1:100
DyLight 405 AffiniPure DonkeyAnti-Rabbit IgG, Jackson ImmunoResearch Laboratories Inc. 711-475-152 N/A 118096 1:100
Donkey anti-Rabbit IgG, Alexa Fluor Â® 555 conjugate, Life Technologies A-31572 N/A 2286312 1:500

Validation

Rabbit anti SoxN was validated by (Ferrero, Fischer and Russell, 2014), and also in this study by lack of staining in null mutant clones.
Rabbit anti-Hth, mouse anti-eyeless was validated in (Li, X. et al. 2013) and in this study by lack of staining in null mutant clones or RNAi clones.
Guinea-pig anti-Run, Rabbit anti-Bsh, Rabbit anti-Slp1, Guinea-pig anti-Slp2, Rabbit anti-D, Guinea-pig anti-Tll, and Guinea-pig anti-Dpn were validated by comparing their staining patterns in the optic lobe to those of previously published antibodies against the same proteins (Hasegawa, E. et al. 2011, Li, X. et al. 2013).
Rabbit anti-Sox102F was validated by lack of staining in mutant clones and by showing the same expression pattern as Sox102F::GFP line (Naidu, V. G. et al. 2020).
Rabbit anti-D from John R. Nambu was validated by (Ma et al., 1998), and in this study by D-RNAi clones.
Rat anti-Dfr was validated by (Hasegawa et al., 2011).
Guinea-pig anti-Kn was validated by (Jinushi-Nakao et al., 2007).
Rabbit anti-Opa was validated by (Koromila et al., 2020).
Rat anti-BarH1 was validated by (Charlton-Perkins et al., 2011).
Literature validating commercial antibodies including sheep anti-GFP, Chick anti-beta Galactosidase, Rat anti-Histone H3 (phospho S28) antibody, rabbit anti-RFP, Mouse anti V5-Tag:DyLight®550, Rat anti-Deadpan, mouse anti-Pros, mouse anti-Repo, mouse anti Cut, mouse anti-Dac, mouse anti-Dap, and mouse anti-Lola-F (Lola zf5) can be found in manufactures' websites.

Animals and other organisms

Policy information about studies involving animals; ARRIVE guidelines recommended for reporting animal research

Laboratory animals

Drosophila melanganster Strains:
SoxNGal4(GMR41H10Gal4) (Pfeiffer et al., 2008) N/A
UAS-RedStinger BDSC Cat# 8547
E(spl)mγGFP (Almeida and Bray, 2005) N/A
E(spl)mγGFP ; SoxNGal4 UAS-RedStinger /TM6B This study N/A
SoxNNC14/CyO BDSC Cat# 9938

y,w, hsFLP, UASCD8GFP; FRT40A tubGal80; tubGal4/TM6B (Lee and Luo, 2001) N/A
UAS-eyRNAi BDSC Cat# 32486
ayGal4: (yw hsFLP; act>y+>Gal4 UAS-GFP / CyO) (Ito et al., 1997) N/A
Dmrt99B::GFP BDSC Cat# 81280
UAS-dmrt99BRNAi BDSC Cat# 31982
FRT82B opa7 (Lee, Stultz and Hursh, 2007) N/A
y,w,hsFLP,UASCD8GFP; ; tubGal4, FRT82B tubGal80 /TM6B (Lee and Luo, 2001) N/A
FRT82B hthP2 /TM6B Richard Mann N/A
UAS-opaRNAi VDRC Cat# 101531
UAS-Dcr2;Dpn-LacZ/CyO; VsxGal4/TM6B (Erclik et al., 2017) N/A
FRT40A slpS37A /SM6-TM6B (Sato and Tomlinson, 2007) N/A
yw hs FLP; act>y+>Gal4 UAS GFP / CyO; UASDCR2/TM6B This work N/A
Erm::V5 (Rives- Quinto et al., 2020) N/A
FRT40A erm1/CyO,GFP (Weng, Golden and Lee, 2010) N/A
UAS-ermRNAi BDSC Cat# 50661
yw, hs-Flp1.22 ;; FRT80B, eyBAC, Ubi-GFP/TM6B,Tb; eyJ5.71 (Li et al., 2013) N/A
hs-Flp1.22;; FRT80B; eyJ5.71/ In(4)ciD (Li et al., 2013) N/A
aprK568-lacZ (Cohen et al., 1992) N/A
UAS-scroRNAi BDSC Cat# 29387
UAS-scroRNAi BDSC Cat# 33890
B-H2::GFP BDSC Cat# 67734
UAS-BarH1RNAi VDRC Cat# 104681
UAS-BarH2RNAi VDRC Cat# 11570
UAS-DRNAi VDRC Cat# 107194
Gcm::GFP BDSC Cat# 38647
gcm-LacZ (P{PZ}gcmrA87/CyO) BDSC Cat# 5445
Gcm2::GFP BDSC Cat# 38646
UAS-tll-miRNA (Lin, Huang and Lee, 2009) N/A
Df(2L)200 FRT40A / Gla, Bc (deletes gcm and gcm2) (Chotard, Leung and Salecker, 2005) N/A
UAS-gcmRNAiGD VDRC Cat# 110539
UAS-gcmRNAiKK VDRC Cat# 2961
lola-T::GFP [flybase: (lola.GR -GFP.FLAG)] BDSC Cat# 38661
lola-K::GFP [flybase: (lola.I-GFP.FLAG)] BDSC Cat# 38662
UASDCR2; optixGal4/CyO (Erclik et al., 2017) N/A
UAS-lolaRNAi-TRiP.JF02254 BDSC Cat# 26714
UAS-lolaRNAi-TRiP.GLV21086 BDSC Cat# 35721
UAS-lolaRNAiKK VDRC Cat# 101925
Nerfin-1::GFP BDSC Cat#: 67385
UAS-nerfin-1RNAi VDRC Cat# 101631
UAS-oazRNAiGD VDRC Cat# 39214
tap::GFP BDSC Cat#: 68188
P{PZ}Oaz[03563] BDSC Cat#: 11338
Scrt::GFP VDRC Cat#: 318073
UAS-oazRNAiKK VDRC Cat# 107061
UAS-hbnRNAi VDRC Cat# 103979
UAS-sbaRNAi VDRC Cat# 101314
UAS-scrtRNAi VDRC Cat# 105201
UAS-Scro-3xHA FlyORF Cat# F000666
UAS-Gcm BDSC Cat# 5446

Wild animals	No wild animals were used in the study.
Field-collected samples	No field collected samples were used in the study.
Ethics oversight	The Institutional Biosafety Committee (IBC) at the University of Illinois Urbana-Champaign approved the study. No ethical concerns as no vertebrate or human subjects were used in this manuscript.

Note that full information on the approval of the study protocol must also be provided in the manuscript.

Flow Cytometry

Plots

Confirm that:

- [x] The axis labels state the marker and fluorochrome used (e.g. CD4-FITC).
- [x] The axis scales are clearly visible. Include numbers along axes only for bottom left plot of group (a 'group' is an analysis of identical markers).
- [x] All plots are contour plots with outliers or pseudocolor plots.
- [x] A numerical value for number of cells or percentage (with statistics) is provided.

Methodology

Sample preparation	For each scRNA-seq experiment, 120 third instar larvae of the genotype E(spl)mγGFP ; SoxNGal4 UAS-RedStinger /TM6B were washed with PBS twice, and with 70% ethanal for 1 min, and with PBS once again. Each of the brains was dissected on ice in complete Schneider's culture medium (Schneider's Insect medium, supplemented with 10% fetal bovine serum, 2% Pen/Strep and 0.02 mg/mL insulin). The dissected brains were directly transferred into a glass dish on ice containing DPBS. The dissection was completed within one hour, and then the supernatant (mainly DPBS) was replaced by 1 mL TrypLE with 1mg/mL collagenase I and 1mg/mL papain. The brains were then incubated for 10 min at 30°C, with gentle shaking at 55 rpm. After removal of the dissociation solution, the brains were carefully washed with complete Schneider's culture medium once and with DPBS twice. The brains were disrupted in 1.4 ml of DPBS with 0.04% BSA by manual pipetting using a P1000, and then 0.4 ml of DPBS with 0.04% BSA was added to make a total volume of 1.8 mL. The cell suspension was filtered through the cell strainer cap into a 5mL BDFalcon FACS tube.
Instrument	FACS sorting was done immediately after on BD FACS ARIA II with gentle settings (85 µm nozzle and low pressure of 20 psi)
Software	BD FACSDiva 8.0.1
Cell population abundance	0.1%
Gating strategy	We used SSC-A/FSC-A to exclude debris, and then single cells were selected based on FSC-A/FSC-W. Live cells were selected based on low DAPI staining (Pacific Blue-A). Control cells without GFP or RFP fluorescence were used to set the threshold on FITC-A(GFP) and PE-A(RFP) so that 0% of control cells are GFP+RFP+. For the sample, selected GFP+RFP+ cells were 1 order of magnitude more fluorescent than other cells on FITC-A, and 2-3 orders of magnitude more fluorescent than other cells on PE-A.

- [x] Tick this box to confirm that a figure exemplifying the gating strategy is provided in the Supplementary Information.
